# Targeted Delivery of siRNA to Transferrin Receptor Overexpressing Tumor Cells via Peptide Modified Polyethylenimine

**DOI:** 10.3390/molecules21101334

**Published:** 2016-10-10

**Authors:** Yuran Xie, Bryan Killinger, Anna Moszczynska, Olivia M. Merkel

**Affiliations:** 1 Department of Pharmaceutical Sciences, Wayne State University, 259 Mack Ave, Detroit, MI 48201, USA; yuran.xie@wayne.edu (Y.X.); bryan.killinger@wayne.edu (B.K.); ei2744@wayne.edu (A.M.); 2 Van Andel Institute, Grand Rapids, MI 49503, USA; 3 Department of Pharmacy, Ludwig-Maximilians Universität München, Butenandtstr. 5-13 (Haus B), D 81377 Munich, Germany

**Keywords:** siRNA delivery, transferrin receptor, targeting, peptide, polyethylenimine

## Abstract

The use of small interference RNA (siRNA) to target oncogenes is a promising treatment approach for cancer. However, siRNA cancer therapies are hindered by poor delivery of siRNA to cancer cells. Transferrin receptor (TfR) is overexpressed in many types of tumor cells and therefore is a potential target for the selective delivery of siRNA to cancer cells. Here, we used the TfR binding peptide HAIYPRH (HAI peptide) conjugated to cationic polymer branched polyethylenimine (bPEI), optimized the coupling strategy, and the TfR selective delivery of siRNA was evaluated in cells with high (H1299) and low TfR expression (A549 and H460). The HAI-bPEI conjugate exhibited chemico-physical properties in terms of size, zeta-potential, and siRNA condensation efficiency similar to unmodified bPEI. Confocal microscopy and flow cytometry results revealed that HAI-bPEI selectively delivered siRNA to H1299 cells compared with A549 or H460 cells. Moreover, HAI-bPEI achieved more efficient glyceraldehyde 3-phosphate dehydrogenase (GAPDH) gene knockdown in H1299 cells compared with bPEI alone. However, despite optimization of the targeting peptide and coupling strategy, HAI-bPEI can only silence reporter gene enhanced green fluorescent protein (eGFP) at the protein level when chloroquine is present, indicating that further optimization of the conjugate is required. In conclusion, the HAI peptide may be useful to target TfR overexpressing tumors in targeted gene and siRNA delivery approaches.

## 1. Introduction

Small interfering RNA (siRNA) is double stranded RNA that silences gene transcription in a sequence specific manner. siRNA can specifically and efficiently silence oncogenic genes, providing a promising alternative for cancer therapy [1]. However, in vivo applications of siRNA face many challenges including enzymatic degradation, aggregation with serum proteins, poor penetration of tissue and crossing biological membranes, non-specific binding, and inefficient endosomal escape [2]. To overcome these challenges, siRNA is commonly formulated with a delivery vector [2,3], which is branched polyethylenimine (bPEI) in the present study. The cationic polymer bPEI, as a nucleic acid vector, has been reported to mediate efficient cellular uptake and endosomal escape both in vitro and in vivo [4]. Polymers modified with receptor specific ligands have been used to efficiently and selectively deliver siRNA to tumor cells [5]. Transferrin receptor (TfR) is a membrane receptor which regulates endocytosis of iron transport protein (Tf), and is expressed at a low level on most cells. However, TfR is overexpressed in many kinds of cancers due to their increased consumption of iron. The upregulation of TfR in cancer cells makes it a promising target for the selective delivery of siRNA for cancer therapies [6]. Several strategies have been explored to target TfR, including the use of holo-Tf or monoclonal antibodies raised against TfR [7,8] which were coupled to polymers [9], and liposomes [10]. However, high concentrations of endogenous Tf in blood plasma (25 µM) may competitively inhibit the uptake mediated by the holo-Tf or the anti-TfR antibody [11]. Furthermore, holo-Tf (molecular weight (MW) 80 kDa) and antibodies against TfR (e.g., the anti-human TfR antibody 5E9, MW = 30 kDa [7]) are relatively large, and therefore, may introduce steric hindrance when the drug delivery system is synthesized. The stability of these proteins will also need to be taken into consideration in terms of synthesis conditions, storage, formulation, and expected shelf-life. A viable alternative to Tf and TfR specific antibodies is the use of TfR binding peptides. Two peptide sequences, HAIYPRH and THRPPMWSPVWP, discovered by phage display, were reported to be able to non-competitively bind to human TfR with high affinity and consequently trigger TfR internalization [12]. The chemico-physical properties of these peptides are more stable and their MWs are smaller than those of holo-Tf and antibodies. 

The aim of this study was to develop a siRNA delivery system to target TfR overexpressing tumor cells. In the present work, two TfR targeting peptides and bPEI were successfully coupled via two different crosslinkers, namely PEGylated succinimidyl 3-(2-pyridyldithio) propionate (PEG_4_-SPDP) and sulfosuccinimidyl 4-(N-maleimidomethyl) cyclohexane-1-carboxylate (sulfo-SMCC). The siRNA and bPEI or peptide-bPEI polyplexes were characterized regarding size, zeta-potential, and siRNA condensation efficiency. Selective delivery of siRNA using peptide-bPEI was investigated in the TfR overexpressing cell line H1299 and low TfR expressing cell lines A549 or H460. Gene knockdown efficiency of siRNA/peptide-bPEI polyplexes was measured both at the mRNA and protein level in H1299 cells.

## 2. Results

### 2.1. Synthesis of HAI-bPEI

**Figure 1:**
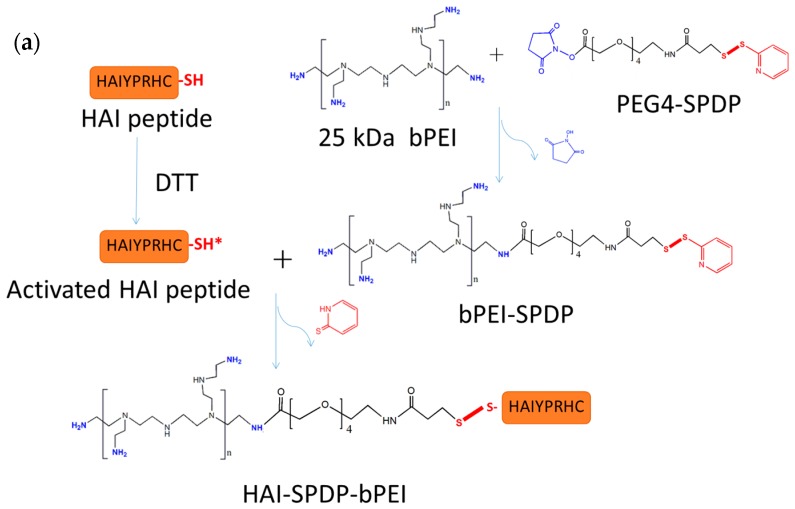

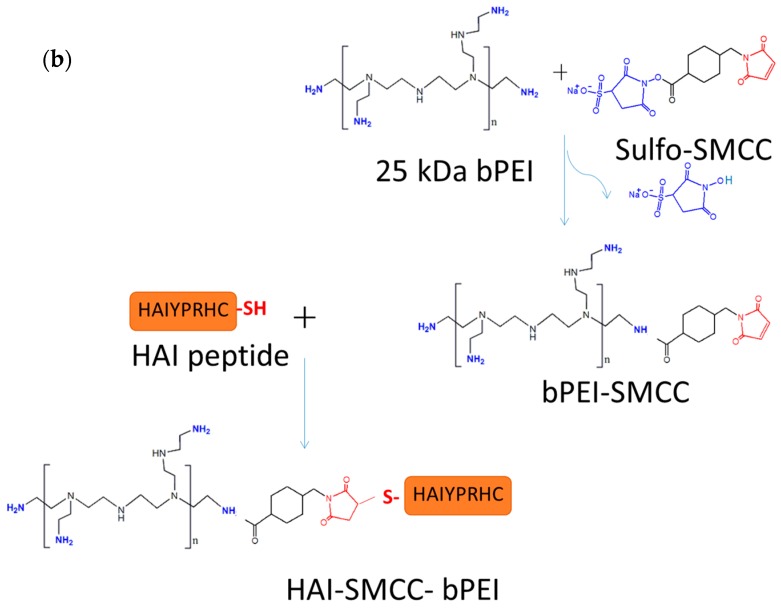
(**a**) Synthesis approach of HAI-SPDP-bPEI.; (**b**) Synthesis approach of HAI-SMCC-bPEI. (bPEI: branched polyethylenimine; PEG_4_-SPDP: 2-Pyridyldithiol-tetraoxatetradecane-N-hydroxysuccinimide; sulfo-SMCC: sulfosuccinimidyl 4-[N-maleimidomethyl]cyclohexane-1-carboxylate; DTT: dithiothreitol).

HAI-bPEI conjugates were successfully synthesized using crosslinkers sulfo-SMCC or PEG_4_-SPDP (Figure 1a,b). To synthesize HAI-SMCC-bPEI, sulfo-SMCC was first reacted with primary amines in bPEI, then the maleimide group in bPEI-SMCC was reacted with sulfhydryl in the cysteine modified HAI peptide. To synthesize HAI-SPDP-bPEI, PEG_4_-SPDP was first coupled with bPEI. After purification, bPEI-PEG_4_-SPDP was coupled to DTT-reduced cysteine-modified HAI peptide. The molar ratios of HAI peptide to bPEI were determined according to the absorbance at 280 nm and the 2,4,6-trinitrobenzene sulfonic acid (TNBS) assay. The molar ratio of HAI peptide to bPEI was 18.6 for HAI-SMCC-bPEI and 18.2 for HAI-SPDP-bPEI.

### 2.2. siRNA Condensation Efficiency of bPEI and HAI-bPEI

**Figure 2:**
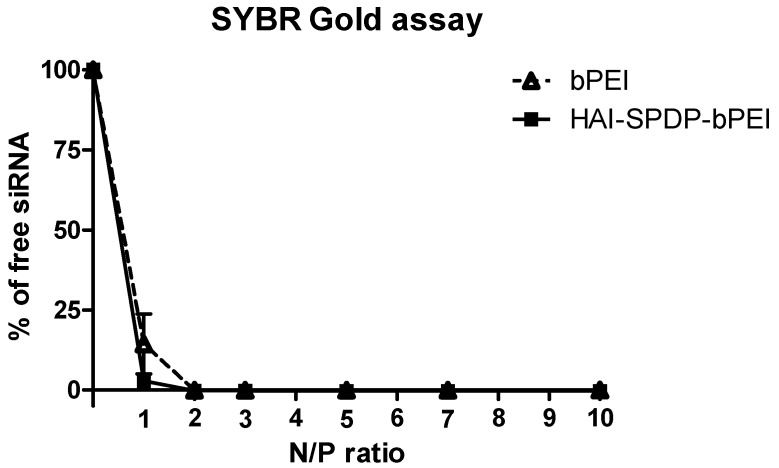
The siRNA condensation efficiency of bPEI and HAI-SPDP-bPEI was determined by a SYBR^®^ Gold assay at N/P ratio 1, 2, 3, 5, 7 and 10. At N/P = 0, the fluorescence represented 100% free siRNA. (Data points indicate mean ± standard deviation (SD), *n* = 3.)

Cationic polymers, such as bPEI, can condense siRNA via ionic interactions. The amount of uncondensed free siRNA can be quantified by SYBR^®^ Gold, an intercalating fluorescent nucleic acid staining dye. Therefore, the condensation efficiency of bPEI and HAI-bPEI at different anime to phosphate (N/P) ratios (1, 2, 3, 5, 7, 10) was examined by a SYBR Gold assay. As shown in Figure 2, bPEI and HAI-SPDP-bPEI demonstrated similar condensation efficiency. Increasing the N/P ratio resulted in less free siRNA. Both bPEI and HAI-bPEI can achieve complete condensation of siRNA at an N/P ratio of 2 and all N/P ratios above 2, respectively.

### 2.3. Hydrodynamic Size and Zeta-Potential of bPEI and HAI-bPEI Polyplexes

**Figure 3:**
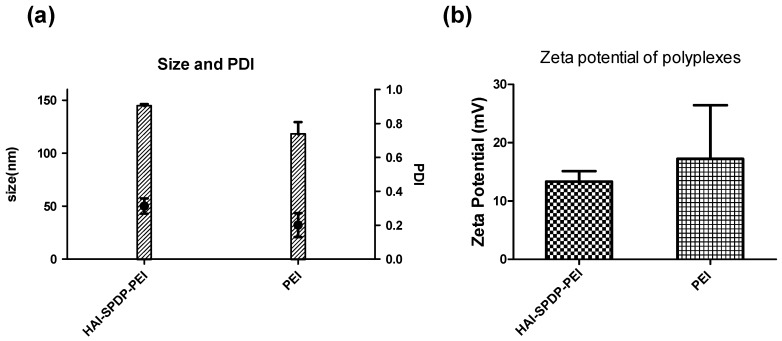
(**a**) Hydrodynamic diameters (left *y*-axis) and polydispersity index (PDI, right *y*-axis) and (**b**) zeta-potential of bPEI and HAI-SPDP-bPEI polyplexes containing 50 pmol of siRNA at N/P = 5. (Data points indicate mean ± SD, *n* = 3.)

The particle size of HAI-SPDP-bPEI and bPEI polyplexes formulated with 50 pmol of siRNA at N/P = 5 was measured by dynamic light scattering (Figure 3a). HAI peptide modified bPEI polyplexes, as expected, had a slightly larger hydrodynamic diameter of around 144 nm with a polydispersity index (PDI) of around 0.3. Unmodified bPEI polyplexes exhibited a hydrodynamic diameter of around 117 nm with a PDI of around 0.2. The zeta-potential of HAI-SPDP-bPEI and bPEI polyplexes were also determined. As shown in Figure 3b, the zeta-potentials of both polyplexes were slightly positive, and HAI-SPDP-bPEI polyplexes demonstrated a lower positive surface charge (13.3 ± 1.7 mV) than bPEI polyplexes (17.2 ± 9.2 mV). 

### 2.4. TfR Expression

**Figure 4:**
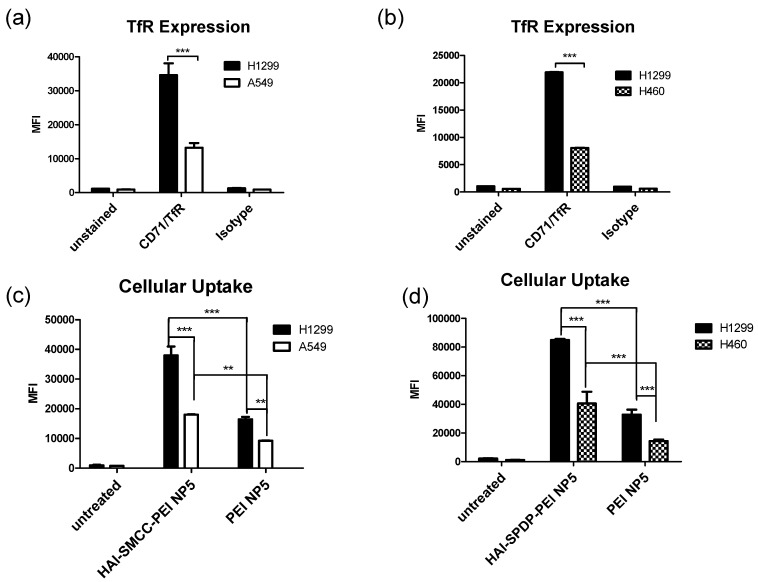
(**a**) H1299 and A549 cells were immunostained by the anti-CD71 antibody which binds to transferrin receptors (TfR) and by the isotype antibody which served as a control of non-specific binding. Median fluorescence intensity (MFIs) were quantified via flow cytometry; (**b**) H1299 and H460 cells were immunostained by the anti- CD71 antibody and by the isotype antibody (Data points indicate mean ± SD, *n* = 2. *** *p* < 0.001); (**c**) The cellular uptake of bPEI and HAI-SPDP-bPEI polyplexes was determined in TfR overexpressing cells H1299 and TfR low expressing cells A549. Polyplexes were prepared with 50 pmol of siRNA fluorescently labeled with Alexa Fluor 488 at N/P = 5 and transfected for 24 h. The MFIs were quantified by flow cytometry. (Data points indicate mean ± SD, *n* = 2. ** *p* < 0.01, *** *p* < 0.001); (**d**) The cellular uptake of bPEI and HAI-SPDP-bPEI polyplexes was determined in TfR overexpressing cells H1299 and TfR low expressing cells H460. (Data points indicate mean ± SD, *n* = 3. *** *p* < 0.001).

TfR expression levels in H1299, H460 and A549 cells were measured via flow cytometry. Cells were immunostained with a fluorescently labeled anti-CD71 antibody, namely the anti-TfR antibody, and with an isotype control antibody. As shown in Figure 4a,b, median fluorescent intensity (MFI) across all three cell lines was unchanged when stained with the isotype antibody, indicating that little fluorescence was caused by non-specific binding of the antibody. On the other hand, H1299 cells demonstrated a significantly higher MFI after being stained with the anti-CD71 antibody compared with A549 and H460 cells. Therefore, H1299 cells were considered as a TfR overexpressing cell model while A549 and H460 cells were considered as TfR low expressing cell models for later studies.

### 2.5. Cellular Uptake of bPEI and HAI-bPEI Polyplexes

The bPEI modified by HAI peptide via two different crosslinkers—sulfo-SMCC or PEG_4_-SPDP, HAI-SMCC-bPEI or HAI-SPDP-bPEI—were tested in this study. The cellular uptake of HAI-SMCC-bPEI or HAI-SPDP-bPEI polyplexes compared with bPEI polyplexes were determined in the TfR overexpressing cell line H1299, and in TfR low expressing cell lines H460 or A549 via flow cytometry. Cells were transfected by HAI-SMCC-bPEI, HAI-SPDP-bPEI or bPEI polyplexes containing siRNA labeled with Alexa Fluor 488 at N/P = 5 for 24 h. As shown in Figure 4c, significantly stronger fluorescence was determined in H1299 cells treated with HAI-SMCC-bPEI polyplexes than in A549 cells. On the other hand, only a slightly stronger MFI was observed in H1299 cells treated with bPEI polyplexes than in A549 cells. In A549 cells, cellular uptake mediated by HAI-SMCC-bPEI polyplexes was only slightly higher than that mediated by bPEI polyplexes, however, the difference between cellular uptake mediated by HAI-SMCC-bPEI and bPEI polyplexes was more significant in H1299 cells. A comparison of the cellular uptake mediated by HAI-SPDP-bPEI and bPEI polyplexes was performed in H1299 and H460, as shown in Figure 4d, and a similar cellular uptake profile was observed.

### 2.6. Imaging of Uptake by Confocal Laser Scanning Microscopy (CLSM)

**Figure 5:**
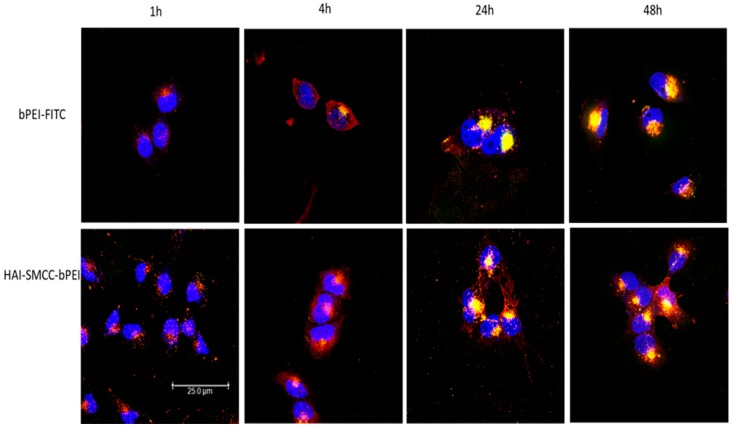
Subcellular distribution of bPEI and HAI-SMCC-bPEI polyplexes in H1299 was imaged by the confocal microscope. H1299 cells were transfected with bPEI and HAI-SMCC-bPEI polyplexes prepared with 50 pmol of siRNA fluorescently labeled with Ty563 at N/P = 5 for 1, 4, 24 and 48 h. The polymers were fluorescently labeled with FITC and shown in green. siRNA-Ty563 was shown in red, and DRAQ5^TM^ stained nuclei were shown in blue.

To determine the sub-cellular distribution of polyplexes, H1299 cells were transfected by HAI-SMCC-bPEI fluorescently labeled by fluorescein isothiocyanate (HAI-SMCC-bPEI-FITC) and bPEI-FITC polyplexes containing siRNA fluorescently labeled with Ty563 at N/P = 5 for 48 h. The cellular distribution of polyplexes was observed at several time-points (1, 4, 24, and 48 h) by CLSM (Figure 5). H1299 cells treated with HAI-SMCC-bPEI polyplexes (Figure 5, lower panel) showed denser distribution of the red signal representing siRNA-Ty563 than cells treated with bPEI polyplexes (Figure 5, upper panel). At 24 and 48 h, bright yellow dots, representing co-localization of polymer-FITC (green) and siRNA-Ty563 (red), were observed in both cells treated with HAI-SMCC-bPEI and bPEI polyplexes. There were some red dots (siRNA-Ty563) evenly distributed in the cytoplasm of cells treated with HAI-SMCC-bPEI polyplexes, however, siRNA-Ty563 was exclusively co-localized with bPEI (yellow dots) and not present as free siRNA in cells transfected with bPEI polyplexes. 

**Figure 6:**
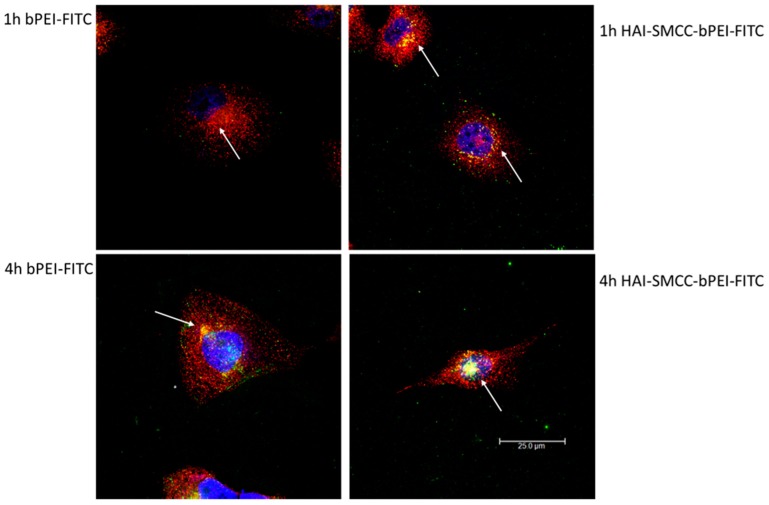
Co-localization of transferrin (Tf) and bPEI or HAI-SMCC-bPEI polyplexes in H1299 was imaged by the confocal microscope. H1299 cells were incubated with Tf fluorescently labeled with Texas Red (Tf-Texas Red) and bPEI or HAI-SMCC-bPEI polyplexes prepared with 50 pmol of siRNA at N/P = 5 for 1 and 4 h. The polymers were fluorescently labeled with FITC and shown in green. Tf-Texas Red was shown in red, and DRAQ5 stained nuclei were shown in blue. The arrow pointing to the yellow dots represents co-localization spots of polyplexes and Tf.

To investigate the pathway of cellular internalization mediated by the HAI peptide, H1299 cells were incubated with 2 µg/mL of transferrin which was fluorescently labeled with Texas Red (Tf-Texas Red) and HAI-SMCC-bPEI-FITC or bPEI-FITC polyplexes formulated with non-fluorescently labeled siRNA at N/P = 5 for 1 and 4 h. As shown in Figure 6, upper panel, after 1 h of incubation, more yellow dots representing co-localization of Tf-Texas Red (red) and polymer-FITC (green) were observed in cells treated with HAI-SMCC-bPEI polyplexes than in bPEI polyplexes transfected cells. At 4 h (Figure 6, lower panel), there was noticeably more co-localization between Tf and polymers in both bPEI and HAI-SMCC-bPEI transfected samples when compared with samples at 1 h (Figure 6, upper panel). The intensity of the co-localization was stronger in samples treated with HAI-SMCC-bPEI polyplexes than in bPEI polyplexes-transfected cells (Figure 6, lower panel).

### 2.7. In Vitro GAPDH Gene Knockdown

**Figure 7:**
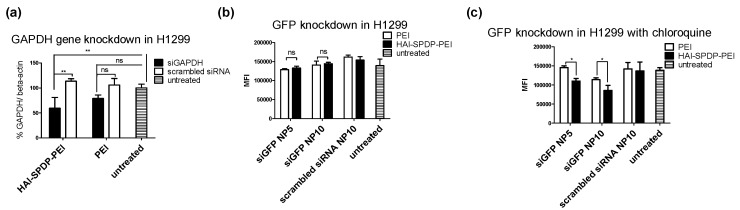
(**a**) H1299 were transfected with bPEI or HAI-SPDP-bPEI polyplexes formulated with 50 pmol of siRNA against GAPDH (siGAPDH) or scrambled siRNA at N/P = 5 for 24 h. The expression of GAPDH was determined by RT-PCR and normalized to the expression of β-actin. Untreated control represented 100% GAPDH/β-actin. (Data points indicate mean ± SD, *n* = 3. No significance (ns), *p* > 0.05, ** *p* < 0.01); (**b**) H1299/eGFP cells were transfected with bPEI and HAI-SPDP-bPEI polyplexes for 48 h without chloroquine treatment; (**c**) H1299/eGFP cells, stably expressing eGFP, were transfected with bPEI or HAI-SPDP-bPEI polyplexes prepared with 50 pmol of siRNA against eGFP (siGFP) or scrambled siRNA at N/P = 5 and 10 for 48 h with chloroquine treatment. The MFIs of eGFP were quantified by flow cytometry. (Data points indicate mean ± SD, *n* = 3. ns, *p* > 0.05, * *p* < 0.05)

To investigate the selective delivery of siRNA with HAI-SPDP-bPEI, we next measured the efficacy of HAI-SPDP-bPEI/siRNA polyplexes to silence GAPDH in H1299 cells. Cells were transfected with either HAI-SPDP-bPEI or bPEI, and with polyplexes containing siRNA against GAPDH (siGAPDH), or scrambled siRNA at N/P = 5 for 24 h. GAPDH gene expression was normalized to β-actin gene expression. As shown in Figure 7a, significantly higher GAPDH silencing was achieved by siGAPDH/HAI-SPDP-bPEI polyplexes compared with the group treated with scrambled siRNA/HAI-SPDP-bPEI polyplexes, and with the untreated group. However, there was no significant difference among siGAPDH/bPEI, the scrambled siRNA/bPEI treated group, and the untreated group.

### 2.8. In Vitro eGFP Knockdown 

To further determine the gene silencing efficiency of HAI-SPDP-bPEI polyplexes at the protein level, the in vitro knockdown efficiency of the reporter gene enhanced green fluorescent protein (eGFP) by HAI-SPDP-bPEI polyplexes was determined in a H1299 cell line stably expressing eGFP. Cells were transfected with HAI-SPDP-bPEI or bPEI polyplexes formulated with siRNA against eGFP (siGFP) or scrambled siRNA at N/P = 5 and 10 for 48 h with or without chloroquine treatment, a drug reported to increase the endosomal release of siRNA [13]. The MFIs of eGFP in cells were quantified by flow cytometry. As shown in Figure 7b, there was no significant difference of MFI between cells transfected with bPEI and HAI-SPDP-bPEI polyplexes containing siGFP without chloroquine treatment at either N/P = 5 or 10. However, with additional chloroquine treatment (Figure 7c), HAI-SPDP-bPEI polyplexes achieved significantly more eGFP knockdown compared with bPEI polyplexes at both N/P = 5 and 10. Both HAI-SPDP-bPEI and bPEI polyplexes containing scrambled siRNA did not reduce the MFI of eGFP, indicating that the eGFP knockdown was not contributed to by the polymer, but by the siRNA against eGFP released in the cytoplasm.

## 3. Discussion

The selective and efficient delivery of siRNA to tumors is hindered by many biological barriers [2]. Numerous modifications of polymers have been discovered to target tumor cells including folic acid [14,15,16], transferrin [17], epidermal growth factor (EGF) [18,19] and the tripeptide arginine-glycine-aspartate RGD [20]. In this study, two human TfR binding peptides, HAIYPRH (HAI peptide) and THRPPMWSPVWP (THR peptide), were tested for gene delivery in TfR positive cells. Both peptides contain a distal cysteine to introduce an additional thiol group for further modification. The THR peptide was first evaluated since it demonstrated a high affinity to TfR (1.5 × 10^−8^ M) compared to the affinity of native ligand Tf to TfR of 2.8 × 10^−9^ M; in contrast, the affinity constant of the HAI peptide was 4.4 × 10^−4^ M [12]. The THR peptide was successfully coupled to 5 kDa bPEI via linker SPDP as shown in Figure S1. Despite the fact that the THR peptide, which was fluorescently labeled with Alexa Fluor 488 (THR-AF488), did preferentially bind to TfR overexpressing H1299 cells compared to TfR low expressing A549 cells (Figure S2a), the cellular uptake mediated by THR-bPEI polyplexes in H1299 cells was lower than that of bPEI polyplexes and showed no difference compared with A549 cells (Figure S2b). It has been reported that the THR peptide conjugated with a chelator for radiolabeling demonstrated negligible uptake in a TfR positive cell line [21] and that THR modified gold nanoparticles did not efficiently cross the blood brain barrier without the addition of a hydrophobic peptide to facilitate membrane binding [22]. Therefore, it is possible that the binding cavity of the THR peptide to TfR is sterically very strict and only allows limited modification in the peptide. Consequently, THR modified with a small fluorescent probe, AF488 (MW = 720.66 Da), still demonstrated preferential binding to H1299 but THR conjugated to a large polymer, bPEI (MW = 5000 Da), cannot mediate significantly higher cellular uptake in H1299 cells compared with A549 cells. Another possible reason of low selective cellular uptake could be low receptor internalization efficiency. Confocal imaging revealed that THR-AF488 can bind to the membrane of H1299 but little internalization was observed (Figure S3), and the co-localization of THR-AF488 and Tf was absent. Thus, THR may not be an optimal candidate for the TfR targeting of polyplexes. 

The other peptide discovered in the same study, the HAI peptide, demonstrated different properties during evaluation for polyplex targeting. Firstly, the HAI peptide was successfully conjugated to 25 kDa bPEI via two different linkers: sulfo-SMCC and PEG_4_-SPDP. The coupling degrees were similar in both approaches and were around 18 HAI peptides per bPEI. It has been reported that protein/peptide modifications in polymer may result in less nucleic acid condensation efficiency compared to the non-modified polymer due to the introduction of steric hindrance [23,24]. The siRNA condensation efficiency of bPEI and HAI-SPDP-bPEI was therefore determined by a SYBR Gold assay, and the results demonstrated that HAI-peptide modification did not have any negative impact on bPEI’s condensation of siRNA (Figure 2). Chemico-physical properties of polyplexes are very important for successful and efficient siRNA delivery. Therefore, bPEI and HAI-SPDP-bPEI polyplexes (N/P = 5) were fully characterized in terms of the hydrodynamic diameter, PDI and zeta-potential. As shown in Figure 3a, HAI-SPDP-bPEI and bPEI polyplexes had narrow size ranges with PDIs of around 0.3, indicating that uniform polyplexes were formed with little aggregation. HAI-SPDP-bPEI polyplexes were slightly larger than bPEI polyplexes which may be due to steric hindrance of the peptide during polyplexes formation. However, both polyplexes resulted in very small sizes (100–150 nm). These results suggested that both polyplexes may readily be taken up by cells [25], avoid rapid clearance from macrophages in the liver and spleen after systemic administration, and could potentially easily accumulate in a tumor through the EPR effect [26]. Both polyplexes demonstrated slightly positive surface charge (<30 mV) (Figure 3b). Since HAI-SPDP-bPEI and bPEI can completely condense siRNA at N/P = 2 (Figure 2), it was expected that at N/P = 5, an excess amount of cationic polymer formulated with siRNA would result in positive surface charges. HAI-SPDP-bPEI polyplexes exhibited a less positive charge than bPEI polyplexes which may be due to the assumption that the conjugated HAI peptide locates on the surface of the polyplexes and can shield some positive charge from bPEI. Another possible reason for the reduced surface charge of HAI-SPDP-PEI is that the SPDP coupling reaction consumed some of the primary amines in bPEI. A slightly positive surface charge could facilitate the binding between polyplexes and cellular membranes. Moreover, the HAI peptide located on the surface is expected to increase the possibility to interact with TfR, suggesting that efficient cellular uptake may be achieved by this formulation.

The TfR expression status of three different cell lines—H1299, A549 and H460—was determined by flow cytometry after immunostaining with the anti-CD71 (TfR) antibody. Based on the results (Figure 4a,b), H1299 cells served as a TfR overexpressing cell model, and A549 and H460 served as TfR low expressing cell models for later studies. The peptide mediated TfR selectivity was investigated by confocal microscopy. HAI-SMCC-bPEI and bPEI were fluorescently labeled with FITC (HAI-SMCC-bPEI-FITC, bPEI-FITC respectively), and polyplexes were formed with non-fluorescently labeled siRNA. H1299 cells were incubated with Tf-Texas Red and the aforementioned polyplexes. As shown in Figure 6, HAI-SMCC-bPEI polyplexes showed more binding (green dots) on the cell surface and more co-localization (yellow dots) with Tf-Texas Red (red dots) than bPEI polyplexes after 1 and 4 h of the co-incubation period, indicating that the HAI peptide can mediate selective binding to TfR overexpressing cells. Furthermore, HAI-SMCC-bPEI can achieve more co-localization with Tf, suggesting that HAI-SMCC-bPEI polyplexes may enter cells via a TfR mediated pathway in a non-competitive manner as reported before [12]. The subcellular distribution of both polyplexes over time was observed under the confocal microscope (Figure 5). H1299 cells were transfected with HAI-SMCC-bPEI-FITC or bPEI-FITC polyplexes (green dots) containing siRNA-Ty563 (red dots). HAI-SMCC-bPEI polyplexes clearly achieved more cellular binding on the membrane than bPEI polyplexes during the first 1 and 4 h, suggesting that HAI-SMCC-bPEI preferentially bound to TfR overexpressing cells compared with bPEI polyplexes. At later time points (24 and 48 h), both bPEI and HAI-SMCC-bPEI polyplexes formed bright yellow dots in vesicle like structures, indicating that many of both types of polyplexes had accumulated and were trapped in endosomes or lysosomes. However, it is worth nothing that more siRNA distributed in the cytoplasm in the HAI-SMCC-bPEI treated group than in bPEI transfected cells. According to the cellular uptake results (Figure 4c), it is possible that HAI-SMCC-bPEI polyplexes mediated higher cellular uptake in H1299 cells than bPEI polyplexes, consequently more siRNA escaped into the cytoplasm. The TfR specific cellular uptake mediated by the HAI peptide was also confirmed by flow cytometry (Figure 4c,d). Both HAI-SMCC-bPEI and HAI-SPDP-bPEI polyplexes showed specific binding to the TfR overexpressing cell line (H1299 cells) compared with TfR low expressing cells (A549 and H460). Furthermore, both HAI peptide modified bPEI polyplexes mediated significantly higher cellular uptake in H1299 cells compared with non-modified bPEI, suggesting a specific interaction between the HAI peptide in the polymer and TfR on the cell membrane.

The gene knockdown efficiency of the HAI peptide, modified bPEI and non-modified bPEI was determined by RT-PCR, and the housekeeping gene GAPDH was targeted. Surprisingly, HAI-SMCC-bPEI polyplexes did not mediate efficient GAPDH gene knockdown, as shown in Figure S4, which did not agree with the cellular uptake result (Figure 4c) and the confocal microscopy result (Figure 5 and Figure 6). It is possible that the HAI peptide modification reduced the number of primary amines in bPEI and consequently reduced the buffering capacity of bPEI. Furthermore, TfR rapidly recycles back to the cell surface after internalization [27], therefore, HAI-SMCC-bPEI polyplexes that are still bound to TfR are recycled back to the cell surface before siRNA can be released. To address this problem, HAI-SPDP-bPEI was synthesized using a linker PEG_4_-SPDP. PEG_4_-SPDP (25.7 Å) is longer than sulfo-SMCC (8.3 Å) which may make the HAI peptide more accessible to TfR and increase the binding efficiency. Moreover, it introduced a disulfide bond into the HAI-SPDP-bPEI conjugate which can be reduced in the endosomal compartment [28] and may facilitate the release of bPEI/siRNA complexes from the HAI peptide/TfR complexes before TfR recycles back to the cell surface. The GAPDH gene knockdown efficiency of HAI-SPDP-bPEI polyplexes was determined in H1299 and H460 cells. In line with previous cellular uptake results (Figure 4d), HAI-SPDP-bPEI polyplexes can achieve more efficient GAPDH gene knockdown in H1299 cells (Figure 7a) compared with bPEI polyplexes but not in H460 cells (Figure S5). These results revealed that the introduction of a long and reducible linker into target moiety-polymer conjugates may increase their transfection efficiency. Additionally, it was shown that the transfection efficiency of HAI-SPDP-bPEI polyplexes is highly dependent on the TfR expression status of the targeted cells.

Next, the gene silencing efficiency of HAI-SPDP-bPEI polyplexes at the protein level was investigated in H1299/eGFP cells. H1299/eGFP cells were treated with HAI-SPDP-bPEI and bPEI polyplexes for 48 h, and the eGFP expression was determined by flow cytometry. However, as shown in Figure 7b, there was no significant difference among the polyplexes treated groups and the untreated group. Based on the confocal images (Figure 5), it is possible that most polyplexes were trapped in the endosome and only a small amount of siRNA was released into the cytoplasm. Therefore, mRNA level silencing can be observed since mRNA is the direct target of siRNA. However, it is more difficult to achieve protein level silencing. A sufficient amount of siRNA needs to be released into the cytoplasm. Additionally, the silencing efficiency also depends on the half-life of the targeted protein, and wild type GFP has a relatively long half-life (around 26 h) [29]. To increase the release of siRNA from endosomes, H1299/eGFP cells were transfected with HAI-SPDP-bPEI and bPEI polyplexes in media containing chloroquine, which is a chemical that can dramatically increase the transfection efficiency by disrupting the endosomal membrane [13]. As shown in Figure 7c, significantly higher eGFP knockdown can be achieved by siGFP/HAI-SPDP-bPEI polyplexes compared with siGFP/bPEI polyplexes in a dose dependent manner. 

## 4. Materials and Methods 

### 4.1. Materials

Branched 5k Da bPEI (Lupasol^®^ G100) and 25k Da bPEI (Lupasol^®^ HF) were obtained from BASF (Ludwigshafen, Germany). PEGylated succinimidyl 3-(2-pyridyldithio) propionate (PEG_4_-SPDP) and sulfosuccinimidyl 4-(N-maleimidomethyl) cyclohexane-1-carboxylate (sulfo-SMCC) were purchased from Thermo Fisher (Waltham, MA, USA). Dulbecco’s phosphate buffered saline (PBS), 1,4-Dithio-DL-threitol (DTT), dimethyl sulfoxide (DMSO), HEPES, ethylenediaminetetraacetic acid disodium salt dehydrate (EDTA), picrylsulfonic acid solution (TNBS, 5% *w/v*), sodium pyruvate, sodium bicarbonate (NaHCO_3_) and glucose were bought from Sigma-Aldrich (St. Louis, MO, USA). Human holo-transferrin was obtained from EMD Millipore (Billerica, MA, USA). Cysteine modified TfR binding peptide HAIYPRH (Cys-His-Ala-Ile-Tyr-Pro-Arg-His) was synthesized by BACHEM Americas (Torrance, CA, USA) and THRPPMWSPVWP (Thr-His-Arg-Pro-Pro-Met-Trp-Ser-Pro-Val-Trp-Pro-Cys) was synthesized by the American Peptide Company (Sunnyvale, CA, USA). Enhanced green fluorescent protein (eGFP) siRNA, amine modified eGFP siRNA, siRNA fluorescently labeled with Ty563 (siRNA-Ty563), human glyceraldehyde-3-phosphate-dehydrogenase (GAPDH) siRNA and scrambled siRNA were purchased from Integrated DNA Technologies (Coralville, IA, USA), the sequence of siRNA see Table 1.

### 4.2. Synthesis of Peptide–bPEI Conjugate

The HAIYPRH peptide (HAI) was coupled to bPEI using two different crosslinkers, namely sulfo-SMCC and PEG_4_-SPDP. In the sulfo-SMCC crosslinking approach, as shown in Figure 1b, 1 mg of 25k bPEI was dissolved in HEPES buffered saline (HBS) buffer (20 mM of HEPES and 150 mM of NaCl, pH = 7.2) at 1 mg/mL, and 1 mg of sulfo-SMCC was dissolved in water at 10 mg/mL. bPEI solution and sulfo-SMCC were mixed together and allowed to stir for 4 h at room temperature (RT). bPEI-SMCC was purified using 10,000 molecular weight cut off (MWCO) centrifugal filters (Millipore) with HBS containing 2 mM of EDTA buffer. The bPEI-SMCC solution was mixed with 1 mg of HAI peptide dissolved in HBS containing 2 mM of EDTA (5 mg/mL) and stirred overnight at RT. The next day, free HAI peptide was removed from HAI-bPEI using 10,000 MWCO centrifugal filters. In the PEG_4_-SPDP crosslinking approach, as shown in Figure 1a, 5 mg of 25k bPEI was dissolved in HBS buffer at 5 mg/mL, and 2 mg of PEG4-SPD was dissolved in DMSO at 20 mM. PEG_4_-SPDP was added drop-wise into the bPEI solution and stirred overnight at RT. Meanwhile, 2 mg of HAI peptide was dissolved in HBS containing 2 mM of EDTA buffer at 4 mg/mL and reduced by 10 molar excess DTT for 2 h. Reduced HAI peptide solution was transferred to a 500–1000 MWCO dialysis tube (Spectrum laboratories, Rancho Dominguez, CA, USA) and dialysis was performed against HBS containing 2 mM of EDTA buffer overnight at 4 °C. The next day, bPEI-SPDP was purified using 10,000 MWCO centrifugal filters with HBS containing 2 mM of EDTA buffer and then mixed with the reduced HAI peptide. The mixture was stirred for 24 h at RT followed by purification using 10,000 MWCO centrifugal filters with HBS. The concentration of HAI peptide in the conjugate was measured spectrophotometrically at 280 nm. The concentration of bPEI in the conjugate was determined by a TNBS assay [30]. Briefly, the according amount of HAI peptide was added into bPEI serial dilution to generate a standard curve in a clear 96-well plate (Corning, Corning, NY, USA). Then 30 µL of 3 mM of TNBS solution was added to 100 µL of bPEI/HAI solution and the absorbance at 405 nm was determined after 5 min.

### 4.3. Fluorescent Labeling of bPEI, Conjugate, Transferrin and siRNA

bPEI was fluorescently labeled with fluorescein-5-isothiocyanate (FITC) (Thermo Fisher). 15 mg of bPEI was dissolved in 0.1 M NaHCO_3_ (pH = 9) at 2.5 mg/mL, and 2 mg of FITC was dissolved in 200 µL of DMSO. The FITC solution was added drop-wise to the bPEI solution and allowed to stir for 3 h at RT. Free FITC was removed using 10,000 MWCO centrifugal filters, and bPEI-FITC was stored in water. The bPEI concentration was determined by a TNBS assay as described above, and the presence of FITC showed no interference with the results, as reported before [20]. For the conjugates, 0.2 mg of FITC (1 mg/mL) in DMSO was added dropwise into 2 mg of HAI-bPEI synthesized via the sulfo-SMCC approach and allowed to react for 3 h with stirring. The concentration of bPEI in the conjugate was determined by a TNBS assay with a bPEI-FITC standard curve. Holo human Transferrin was fluorescently labeled with Texas Red modified with succinimidyl ester (Thermo Fisher) following the manufacturer’s protocol. Amine modified siRNA was fluorescently labeled with Alexa Fluor 488 (Thermo Fisher) following the manufacturer’s protocol, followed by purification using ethanol precipitation and silica spin column binding as reported before [31].

### 4.4. Preparation of Polyplexes

To prepare the siRNA and polymer polyplexes, polymer was diluted with 5% glucose to different concentrations. An equal volume of polymer solution was added to a predetermined amount of siRNA solution to yield different amine to phosphate ratios (N/P ratios) and allowed to incubate for 15 min at RT before measurement or transfection. The calculation to prepare polyplexes is based on the following equation: *m_polymer_* (pg) = *n_siRNA_* (pmol) × *MW_protonabl_*_e_ (g/mol) × *N/P* × *N_nucleotide_* (*MW_protonable_* of bPEI = 43.1 g/mol, *N_nucleotide_* of siRNA = 52).

### 4.5. Measurement of Hydrodynamic Size and Zeta-Potential

Polyplexes were prepared with bPEI or HAI-SPDP-bPEI and 50 pmol of siRNA at N/P 5 in 100 µL 5% glucose buffer as described above and were transferred to a disposable micro cuvette (Brand GMBH, Wertheim, Germany). Size measurement was performed in three runs using a Zetasizer Nano ZS (Malvern Instruments Inc., Westborough, MA, USA). Results were collected and analyzed with the Zetasizer Software (Malvern) with settings of 173° backscatter angle, 0.88 mPa*s for viscosity and 1.33 for refractive index. Subsequently, the same polyplex solutions were diluted to 1 mL with water and transferred to a folded capillary cell (Malvern). The zeta-potentials of polyplexes were determined in three runs using a Zetasizer Nano ZS (Malvern). 

### 4.6. siRNA Condensation Measured by a SYBR Gold Assay

SYBR Gold is a fluorescent dye which is used to stain nucleic acids. It intercalates into siRNA and produces fluorescence. When siRNA is condensed by polymer and becomes inaccessible for SYBR Gold, the fluorescence will decrease. Polyplexes were prepared with bPEI or HAI-SPDP-bPEI and 50 pmol of siRNA at different N/P ratios (1, 2, 3, 5, 7, 10) in 100 µL per well in a FluoroNunc 96 well white plate (Thermo Fisher). Subsequently, 30 µL of 4 × SYBR Gold was added to each well and incubated for 10 min in the dark. The fluorescence of SYBR Gold was determined by a Synergy 2 multi-mode microplate reader (BioTek Instrument, Winooski, VT, USA) with an excitation filter 485/20 nm and an emission filter 520/20 nm. The fluorescence of free siRNA (N/P = 0) represented 100% free siRNA. The experiment was performed in triplicate. 

### 4.7. Cell Culture

NCI-H1299 cells (human non-small cell lung carcinoma) and A549 cells (human non-small cell lung carcinoma) were purchased from ATCC (Manassas, VA, USA). NCI-H460 cells (human large cells lung carcinoma) were a kind gift from Dr. Larry H. Matherly (School of medicine, Wayne State University, Detroit, MI, USA). H1299/eGFP is a H1299 cell line stably expressing the reporter gene eGFP. H1299 and H460 cells were cultured in RPMI-1640 media (GE healthcare, Buckinghamshire, UK) supplemented with 10% heat inactivated fetal bovine serum (*v/v*) (Sigma), 1× penicillin/streptomycin (Corning), 1× GlutaMax (Thermo Fisher), 10 mM of HEPES, 1.5 g/L of NaHCO_3_, 4.5 g/L of glucose and 1 mM of sodium pyruvate. A549 cells were cultured in DMEM media (Corning) supplemented with 10% fetal bovine serum (FBS) and 1× pen/strep. All cells were maintained in a humidified atmosphere and 5% CO_2_ at 37 °C.

### 4.8. Immunofluorescent Staining

H1299, H460 and A549 cells were harvested from flasks and washed with PBS. Each sample contained 100,000 cells and was resuspended in 10 µL of a 50-fold diluted human Fc receptor binding inhibitor (eBioscience, San Diego, CA, USA) for 5 min on ice. Subsequently, 20 µL of a 20-fold diluted phycoerythrin (PE) labeled human CD71 antibody (OKT9, eBioscience) or 20 µL of a 20-fold diluted PE labeled mouse IgG1 isotype control antibody (P3.6.2.8.1, eBioscience) were added to the samples and incubated for 30 min in the dark at 4 °C. Samples were washed with PBS/ 2 mM of EDTA three times and were resuspended in 400 µL of PBS/ 2 mM of EDTA. The median fluorescent intensity (MFI) of samples was quantified using an Attune^®^ Cytometer (Life Technologies, Waltham, MA, USA) with an excitation laser 488 nm and emission filter 574/26 nm. Cell populations were gated according to the forward and side scattering channel, and 10,000 events were collected. Data analysis was performed using the Attune^®^ cytometer software (Life Technologies). Experiments were conducted in duplicate.

### 4.9. Quantification of Cellular Uptake

For cellular uptake experiments, H1299, H460 and A549 cells were seeded at the density of 50,000 cells/well in 24-well plates (Corning). After 24 h of incubation, polyplexes were prepared with 50 pmol of siRNA-AF488 and bPEI, HAI-SMCC-bPEI or HAI-SPDP-bPEI at N/P = 5. The cells were transfected with polyplexes at the siRNA concentration of 125 nM for 4 h. To reduce the potential cytotoxicity induced by concentrated polyplexes, fresh media were added to dilute siRNA concentration to 50 nM followed by an additional 20 h of incubation. The cells were harvested and washed three times with PBS/2 mM of EDTA. All samples were resuspended in 400 µL of PBS/2 mM of EDTA, and MFIs of samples were determined via an Attune^®^ Cytometer (Life Technologies) with an excitation laser 488 nm and emission filter 530/30 nm. In each sample, 10,000 events were collected and data analysis was performed using the Attune^®^ cytometer software (Life Technologies). Experiments were conducted in triplicate. 

### 4.10. Confocal Laser Scanning Microscopy

H1299 cells were seeded at the density of 50,000 cells/well in 24-well plates with a 12 mm circle cover glass (Fisherbrand, 12CIR-1) in each well and were incubated for 24 h. To determine intracellular distribution of polyplexes, fluorescently labeled polymer bPEI-FITC and HAI-SMCC-bPEI-FITC were formulated with 50 pmol of siRNA-Ty563 at N/P = 5. Cells were transfected with polyplexes at a siRNA concentration of 125 nM for the first 4 h and 50 nM for an additional 44 h. Cells were washed with PBS and fixed with 4% paraformaldehyde (PFA) solution in PBS (Affymetrix, Thermo Fisher) for 20 min at RT at different transfection time points (1, 4, 24, 48 h). To investigate the co-localization of the HAI peptide binding part with TfR, cells were incubated with 2 µg/mL of Tf-Texas Red and polyplexes formulated with bPEI-FITC or HAI—SMCC-bPEI-FITC and 50 pmol of siRNA at N/P = 5 for 1 h or 4 h. Slides were washed with PBS and fixed with 4% PFA. After fixation, the nuclei were stained with 5 µM of DRAQ5 (Invitrogen, Thermo Fisher) for 5 min and rinsed with PBS. Slides were mounted with a Fluoromount mounting medium (Southern Biotech, Birmingham, AL, USA). Slides were imaged by a Leica TCS SPE-II laser scanning confocal microscope (Leica, Wetzlar, Germany), and the images were exported from the Leica Image Analysis Suite (Leica).

### 4.11. In Vitro GAPDH Gene Knockdown

To determine mRNA level knockdown efficiency of polyplexes, silencing of housekeeping gene GAPDH in H1299 and H460 was determined by real time PCR (RT-PCR). Cells were seeded at the density of 50,000 cells/well in 24-well plates for 24 h. bPEI or HAI-bPEI were formulated with 50 pmol of siRNA against GAPDH or scrambled siRNA at N/P = 5. Cells were transfected with polyplexes at a siRNA concentration of 125 nM for the first 4 h and 50 nM for an additional 20 h. Total mRNA was isolated from cells using the PureLink^®^ RNA mini kit (Life Technologies) following the manufacturer’s protocol in the addition of DNase I digestion (Sigma). The brilliant III ultra-fast SYBR^®^ green QRT-PCR master mix kit (Agilent Technologies, Santa Clara, CA, USA) was used to reverse transcribe 100 ng of mRNA to cDNA and perform RT-PCR. RT-PCR was conducted in a Stratagene Mx 3005p qPCR system (Agilent Technologies) and cycle threshold (Ct) values were exported from MxPro software (Agilent Technologies). Hs_GAPDH_2_SG primers for GAPDH and Hs_ACTB_2-SG primers for β-actin (Qiagen, Valencia, CA, USA) were used in this experiment. GAPDH gene expression was normalized to β-actin gene expression for quantification and comparison, and the untreated group represented 100% GAPDH expression. Experiments were conducted in triplicate.

### 4.12. In Vitro eGFP Knockdown

To determine the protein level knockdown efficiency of polyplexes, silencing of reporter gene eGFP was determined by flow cytometry. H1299/eGFP cells were seeded at the density of 50,000 cells/well in 24 well-plates for 24 h. On the day of transfection, old media were replaced with 350 µL of fresh media with or without 115 µM of chloroquine. Cells were transfected with 50 pmol of siRNA against eGFP formulated with bPEI or HAI-bPEI at N/P = 5, 10 in total volume of polyplexes of 50 µL. bPEI and HAI-bPEI polyplexes containing scrambled siRNA at N/P = 10 were included as the negative control. Cells were transfected with 50 µL of polyplexes and incubated for 4 h with or without 100 µM of chloroquine. To dilute the concentration of chloroquine and polyplexes, 600 µL of fresh media were added, and cells were incubated for an additional 44 h. Afterward, cells were harvested and washed three times with PBS/2 mM of EDTA. Samples were resuspended in 400 µL of PBS/2 mM of EDTA, and the MFIs were determined by an Attune^®^ Cytometer (Life Technologies) with an excitation laser 488 nm and emission filter 530/30 nm. In each sample, 10,000 events were collected, and data analysis was performed using Attune^®^ cytometer software (Life Technologies). Experiments were conducted in triplicate. 

### 4.13. Statistics

Results were represented as mean ± standard deviation (SD). All statistical analysis used One-way ANOVA with Newman–Keuls multiple comparison post-test in GraphPad Prism software (Graph Pad Software, La Jolla, CA, USA).

## 5. Conclusions

In conclusion, our optimized HAI-bPEI conjugate has desired chemico-physical properties as a siRNA delivery vector. It can achieve selective delivery of siRNA to TfR overexpressing tumor cells and efficient gene knockdown at the mRNA level. This study demonstrates the feasibility to target TfR using HAI peptide. However, results also indicate the need for further optimization of this conjugate to achieve better endosomal escape. 

## Figures and Tables

**Table 1:** Sequence of siRNA.

Name	Sequence
eGFP siRNA	5´-pACCCUGAAGUUCAUCUGCACCACcg 3´-ACUGGGACUUCAAGUAGACGUGGUGGC
GAPDH siRNA	5´- pGGUCGGAGUCAACGGAUUUGGUCgt 3´UUCCAGCCUCAGUUGCCUAAACCAGCA
Scramble siRNA	5´-pCGUUAAUCGCGUAUAAUACGCGUat 3´CAGCAAUUAGCGCAUAUUAUGCGCAUAp

Indication of modified nucleotides: “p” denotes a phosphate residue, lower case bold letters are 2′-deoxyribonucleotides, capital letters are ribonucleotides, and underlined capital letters are 2′-O-methylribonucleotides.

## Supplementary Materials

Supplementary materials can be accessed at: http://www.mdpi.com/1420-3049/21/10/1334/s1.
