# Supplementary material for: Targeted Delivery of siRNA to Transferrin Receptor Overexpressing Tumor Cells via Peptide Modified Polyethylenimine
Source: Molecules. 2016 Oct 10;21(10):1334. doi: 10.3390/molecules21101334 (PMC6273023; doi:10.3390/molecules21101334)
Supplement: Supplementary file 1 [file molecules-21-01334-s001.pdf]

Supplementary Materials: Targeted Delivery of siRNA to Transferrin Receptor Overexpressing Tumor Cells via Peptide Modified Polyethylenimine

Yuran Xie [1], Bryan Killinger, Anna Moszczynska and Olivia M. Merkel

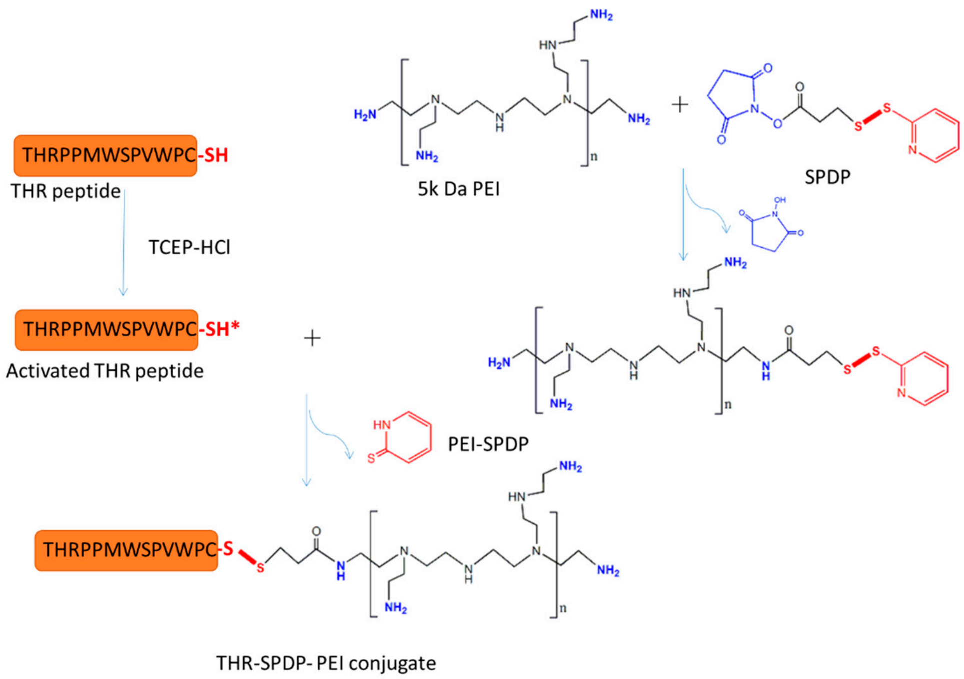

Figure S1. Synthesis approach of THR-SPDP-bPEI. 5k bPEI (1 mg/mL) was dissolved in HBS buffer and mixed with 100 µL 20 Mm of SPDP, stirred overnight, followed by purification via 3000 MWCO centrifugal filters. THRPPMWSPVWP peptide (THR) peptide was reduced by 10-fold excess TCEP (Thermo Fisher) and purified using PD10 column (GE healthcare). THR peptide were mixed with bPEI-SPDP overnight with stirring at RT. The concentration of THR peptide and bPEI in conjugate were measured spectrophotometrically at 280 nm and by a TNBS assay, respectively. (SPDP: succinimidyl 3-(2-pyridyldithio) propionate; TCEP: Tris (2-carboxyethyl) phosphine hydrochloride.)

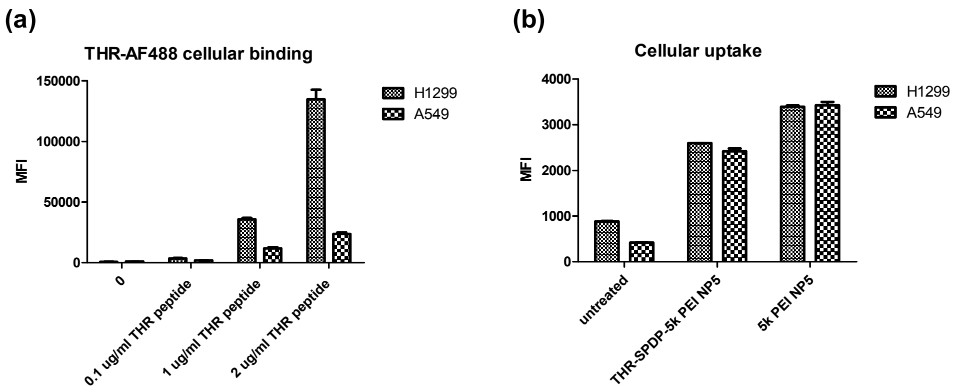

Figure S2. (**a**) Cysteine modified THR peptide were fluorescently labeled by Alexa Fluor 488- maleimide (Thermo Fisher) following the manufacturer's protocol (THR-AF488). Different concentrations of THR-AF488 were incubated with H1299 and A549 for 1 h at 37 °C. The cellular binding of THR-AF488 were quantified by flow cytometry. (Data points indicate mean ± SD, n = 3) (**b**) The cellular uptake of bPEI and THR-bPEI polyplexes was determined in H1299 and A549 cells. Polyplexes were prepared with 50 pmol of siRNA-AF488 at N/P = 5 for 24 h. The cellular uptake was quantified by flow cytometry. (Data points indicate mean ± SD, n = 2–3).

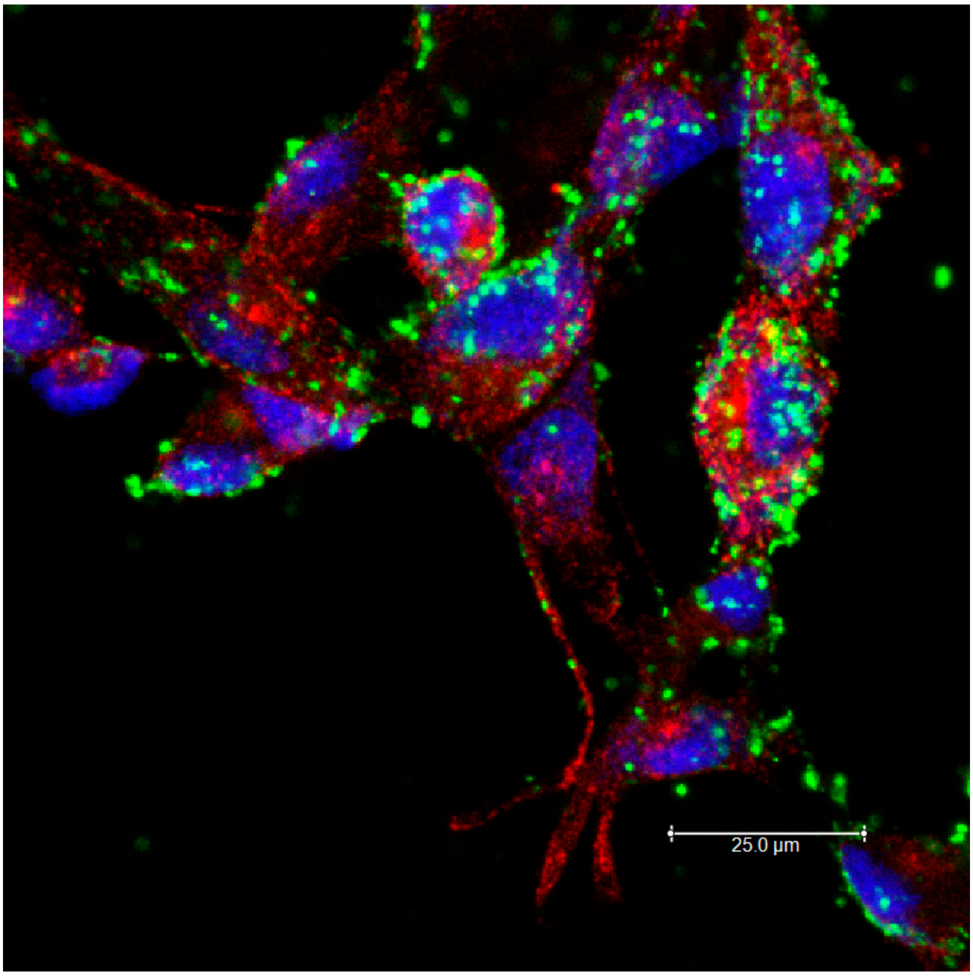

Figure S3. 2 µg/mL of THR-AF488 and Tf-Texas Red were incubated with H1299 for 1 h at 37 °C. The cellular distribution of THR-AF488 and Tf-Texas Red was observed under confocal laser scanning microscopy (CLSM).

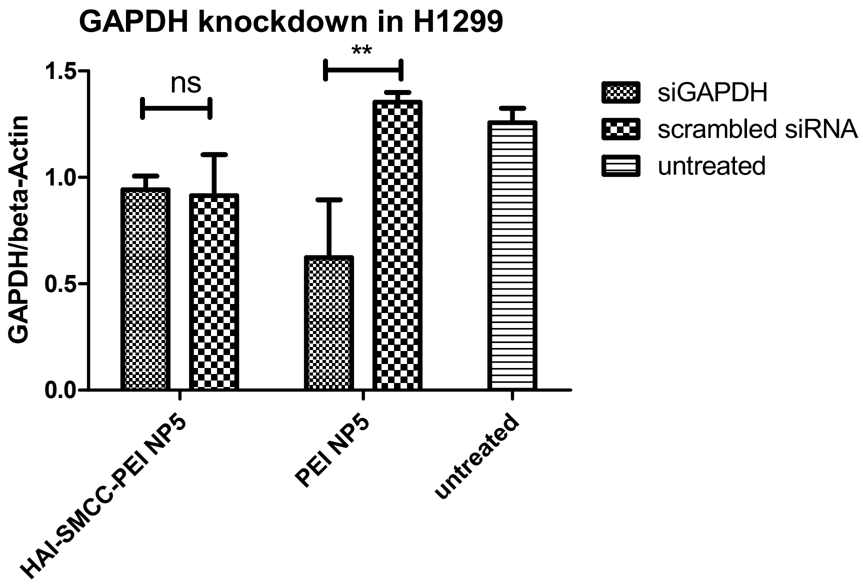

Figure S4. H1299 were transfected with bPEI or THR-bPEI polyplexes formulated with 50 pmol of siGAPDH or scrambled siRNA at N/P = 5 for 24 h. The expression of GAPDH was determined by RT-PCR and normalized to the expression of β-actin. (Data points indicate mean ± SD, n= 3. ns, $p > 0.05$, ** $p < 0.01$).

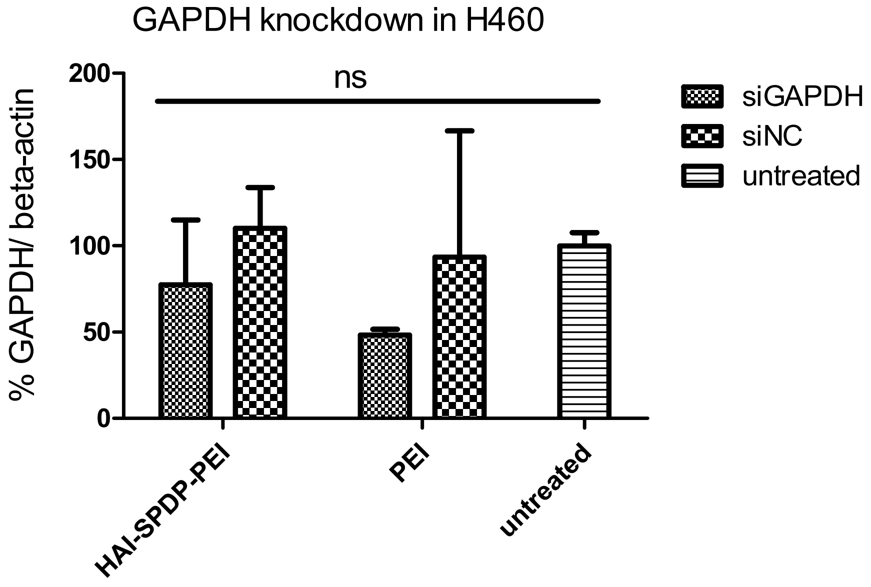

Figure S5. H460 cells were transfected with bPEI or HAI-SPDP-bPEI polyplexes. The expression of GAPDH was determined by RT-PCR and normalized to the expression of β-actin. Untreated control represented 100% GAPDH/β-actin. (Data points indicate mean ± SD, $n = 3$. ns, $p > 0.05$).
